# P-751. A 10-year retrospective review of Necrotizing Fasciitis at a Tertiary Referral Hospital in a Low/Middle Income Country

**DOI:** 10.1093/ofid/ofae631.947

**Published:** 2025-01-29

**Authors:** Tamara Abdallah, Caren Challita, Christopher Abi Zeid Daou, Tania Sawaya, Ali Hallal, Naji Madi, Nesrine Rizk

**Affiliations:** American University of Beirut, Beirut, Beyrouth, Lebanon; American University of Beirut Medical Center, Beirut, Beyrouth, Lebanon; American University of Beirut, Beirut, Beyrouth, Lebanon; American University of Beirut, Beirut, Beyrouth, Lebanon; American University of Beirut, Beirut, Beyrouth, Lebanon; American University of Beirut, Beirut, Beyrouth, Lebanon; American University of Beirut, Beirut, Beyrouth, Lebanon

## Abstract

**Background:**

Necrotizing fasciitis (NF) is a rare, yet potentially fatal skin and soft tissue infection, with a global annual incidence of 0.3 to 15 per 100,000 cases. Early surgical debridement is crucial as mortality rates can reach up to 76%. there is a notable scarcity of data on the epidemiology of NF in the Eastern Mediterranean region. Our aim is to investigate clinical characteristics and outcomes among patients diagnosed with necrotizing fasciitis in a tertiary referral center in Beirut, Lebanon.

Demographic and Clinical Characteristics in the Study Population

**Figure:**
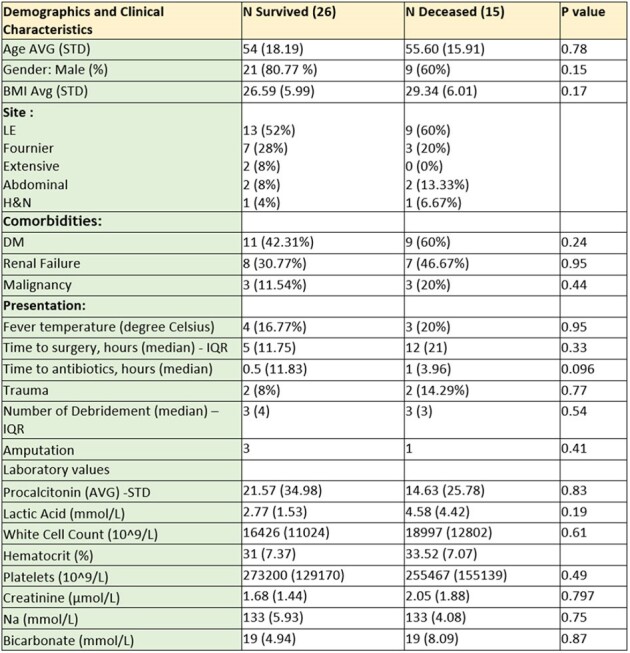

**Methods:**

A retrospective chart review of hospitalized cases between 2011 and 2021. The study population included adult patients diagnosed with NF during this period. Data included demographic and clinical characteristics.

Physical Findings

**Figure:**
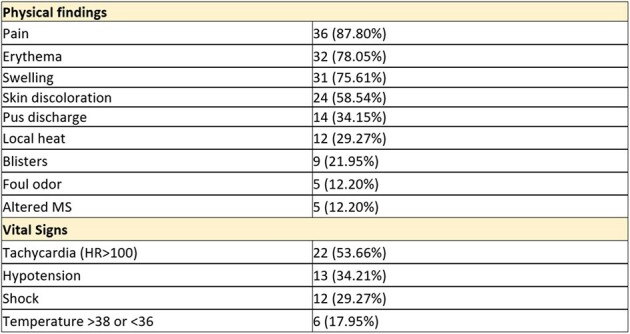

**Results:**

We identified and included a total of 41 cases of NF in this cohort. The average age of the patients was 54.6 years, with males comprising the majority at 73%. The most prevalent co-morbidity was Diabetes Mellitus (48.78%), followed by hypertension (41.46%), cardiovascular diseases (41.46%), and renal failure (36.59%). Pain (87.80%), erythema (78.80%), and swelling (75.61%) were the most common symptoms. Skin discoloration was present in twenty-four of the forty-one patients (59%). Regarding outcomes, the patients identified were divided in two groups: survivors and deceased. overall mortality rate was 37%, and amputation rate was 10% (Table 3). Patients in both groups underwent a median of 3 surgical debridement’s. Time to diagnosis did not significantly differ between survivors (median 1.75 hours) and non-survivors (median 6 hours), whereas antibiotics were administered with a median time of 30 minutes from diagnosis in the survival group, compared to one hour in the deceased group. Time to surgery showed a 7-hour delay in the deceased group compared to survivors (p=0.096).

Timelines and Clinical Outcomes

**Figure:**
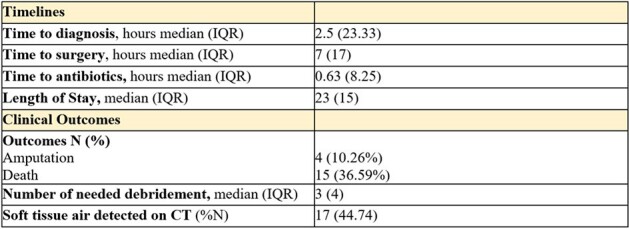

**Conclusion:**

To our knowledge, this study represents the first comprehensive review of necrotizing fasciitis (NF) in Lebanon, providing valuable insights into demographics and comorbidities. Our findings reveal a mortality rate of 37%, which is consistent with the ranges reported in existing literature, highlighting the need for continued efforts to improve outcomes for patients with NF in our region.

**Disclosures:**

**All Authors**: No reported disclosures

